# Successful management of cutaneous lymphangitis carcinomatosa arising from cervical cancer with paclitaxel-cisplatin and bevacizumab combination therapy: a case report and review of the literature

**DOI:** 10.1186/s13256-019-2262-x

**Published:** 2019-11-07

**Authors:** Fumihiro Nakamura, Manabu Seino, Yuriko Suzuki, Hirotsugu Sakaki, Takeshi Sudo, Tsuyoshi Ohta, Seiji Tsutsumi, Satoru Nagase

**Affiliations:** Department of Obstetrics and Gynecology, Yamagata University, School of Medicine, 2-2-2 Iidanishi, Yamagata, Yamagata 990-9585 Japan

**Keywords:** Cervical cancer, Cutaneous lymphangitis carcinomatosa, Skin metastasis, Bevacizumab

## Abstract

**Background:**

Globally, cervical cancer is the fourth most common cancer in women. Here, we report a case of cutaneous lymphangitis carcinomatosa arising from cervical cancer, an extremely rare and treatment-resistant condition.

**Case presentation:**

A 64-year-old Japanese woman presented with genital bleeding. She was diagnosed as having stage IB1 squamous cell cervical cancer and subsequently treated with radiotherapy. Approximately 2 years after the curative radiotherapy, she developed itching, skin rash, and small nodules on her left femoral and pubic area. Slight ^18^F-fluorodeoxyglucose uptake was detected at her left femoral skin on positron emission tomography with computed tomography. A histopathological examination was performed on a biopsy sample from an erythematous macule on her left femoral skin and vulva. Consequently, she was diagnosed as having cutaneous lymphangitis carcinomatosa arising from cervical cancer. Paclitaxel (135 mg/m^2^), cisplatin (50 mg/m^2^), and bevacizumab (15 mg/kg) combination therapy was administered every 21 days. Both itching and rash improved after three treatment cycles. After the completion of six cycles, skin erythema in the femoral and vulval area disappeared completely. Our patient experienced a 25-month symptom-free interval after the last chemotherapy session.

**Conclusion:**

Our findings suggest that combination chemotherapy plus bevacizumab is an effective therapeutic option in patients with cutaneous lymphangitis carcinomatosa arising from cervical cancer.

## Introduction

Cervical cancer is the fourth most common cancer in women worldwide [1], resulting in approximately 275,000 deaths per year [2]. The incidence and mortality rates of cervical cancer in Japan were 16.1 and 4.4 per 100,000 people, respectively. The recurrence rate of cervical cancer was 8–26%, and overall survival after recurrence ranged from 7 to 12 months [2].

The common sites of recurrence are local, lung, liver, bone, and lymph node metastasis [3]. In contrast, cutaneous metastasis, especially cutaneous lymphangitis carcinomatosa arising from cervical cancer, is extremely rare. It is caused by occlusion of the lymphatic channels of the dermis by cancer cells [4]. Skin metastasis, including cutaneous metastasis due to lymphangitis carcinomatosa, is associated with a poor prognosis. The average survival period after the appearance of skin metastasis is as short as 3 to 8.5 months [5–8].

The treatment for recurrent cervical cancer depends on the site of recurrence (local or distant recurrence). Systemic chemotherapy is chosen for the treatment of distant recurrence or local recurrence within the irradiation field. In addition, bevacizumab (BV) has been approved for targeted therapy in patients with recurrent or advanced cervical cancer on the basis of the results of the Gynecologic Oncology Group (GOG) 240 trial [9]. BV is a recombinant humanized monoclonal antibody that inhibits vascular endothelial growth factor (VEGF)-A [10]. VEGF-C, a subfamily within the VEGF family, is expressed in human cancers. VEGF-C promotes tumor angiogenesis and lymphangiogenesis *in vivo*, and drives tumor growth and metastasis [11, 12]. BV has potential antitumor effects against metastatic lesions in the lymph system.

Here, we report a case of cutaneous lymphangitis carcinomatosa arising from cervical cancer that was successfully treated with paclitaxel-cisplatin and BV (TP + BV) combination therapy. We believe that TP + BV therapy can be effective against lymphangitis carcinomatosa arising from cervical cancer.

## Case presentation

**Fig. 1:**
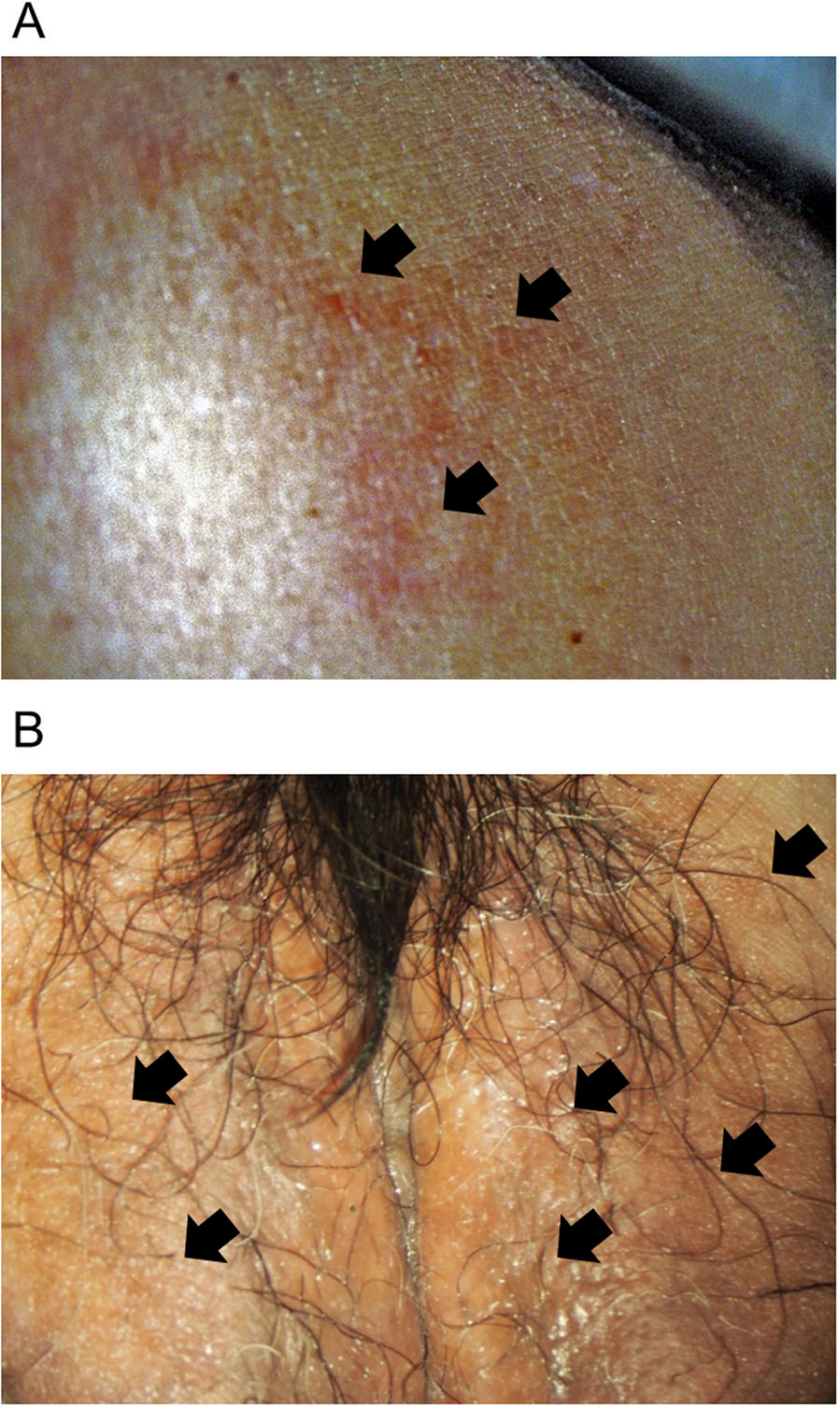
A picture of the patient’s skin. **a** Skin erythema at the left proximal femoral lesion (*arrows*). **b** Small nodules on the vulva (*arrows*)

**Fig. 2:**
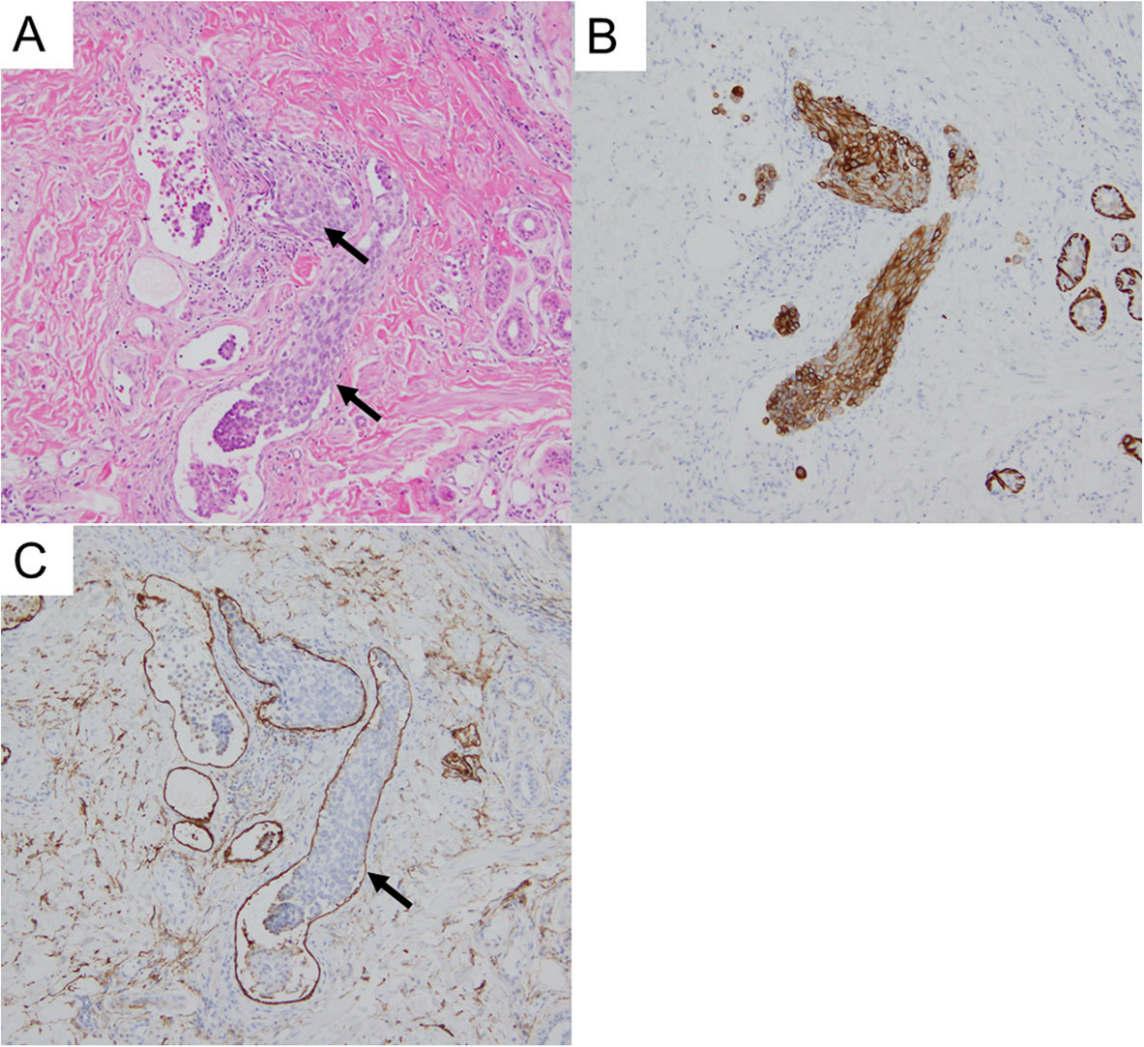
Histopathological analysis. **a** Atypical cells (arrows) similar to the cells in squamous cell carcinoma inside thin-walled vessels (hematoxylin and eosin staining). **b** Atypical cells positive for cytokeratin. **c** Thin-walled vessels positive for D2-40 (arrow)

**Fig. 3:**
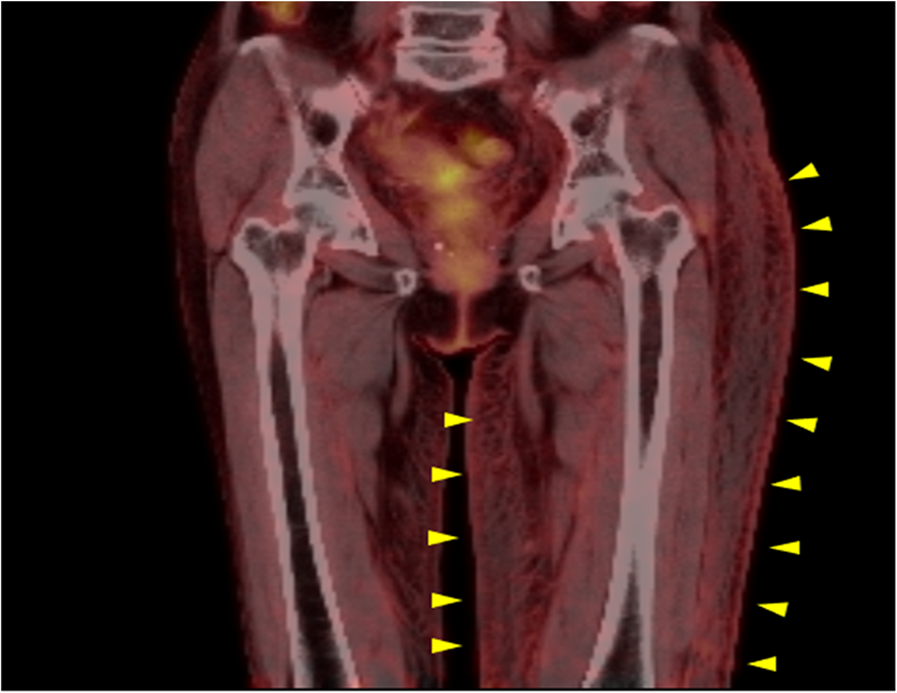
Uptake of ^18^F-fluorodeoxyglucose on positron emission tomography with computed tomography. Slight uptake of ^18^F-fluorodeoxyglucose by the left femoral skin on positron emission tomography with computed tomography (*arrows*)

**Fig. 4:**
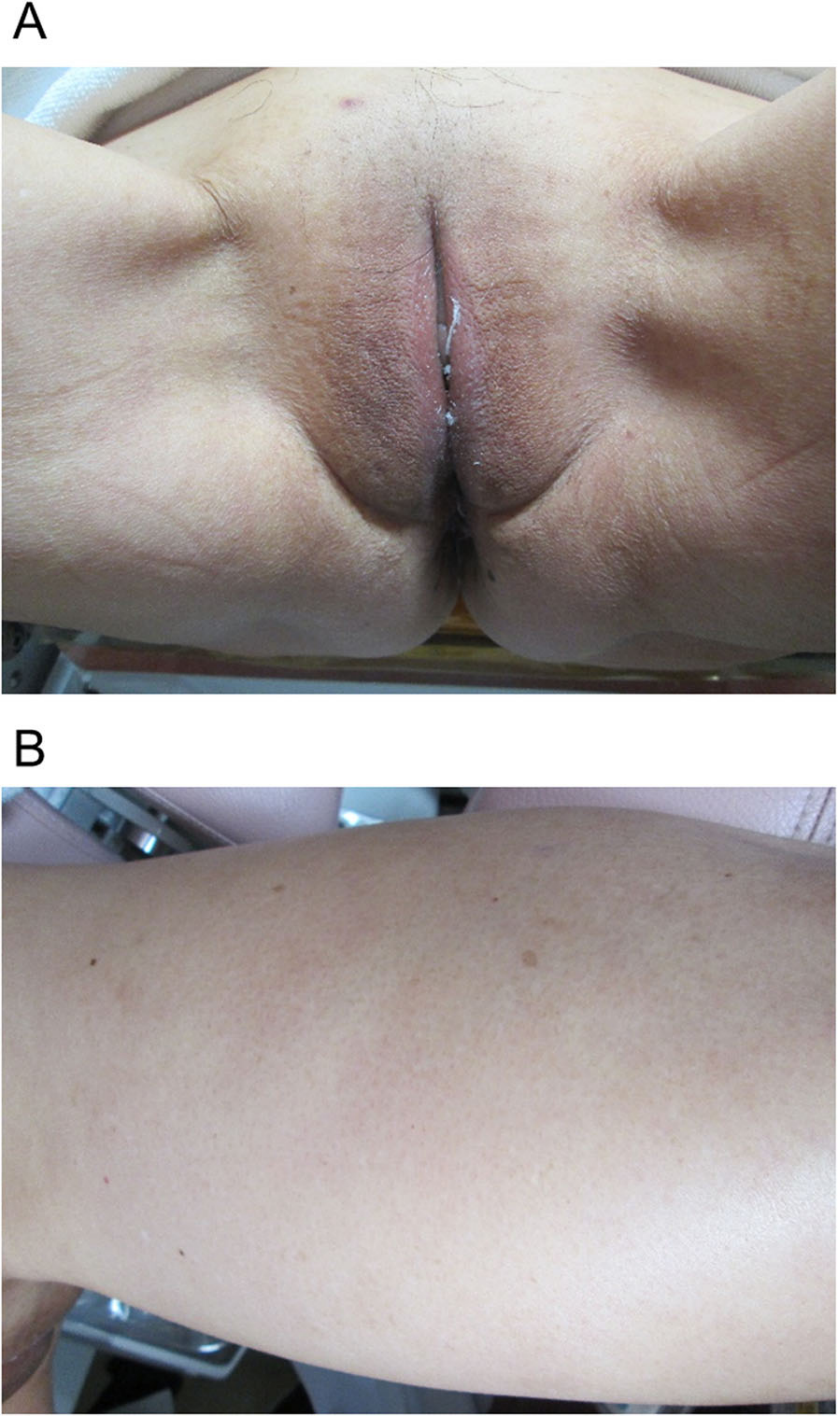
Images acquired after six courses of paclitaxel-cisplatin and bevacizumab combination therapy. **a** Vulva; **b** femoral skin

Our patient was a 64-year-old Japanese woman, gravida 2, para 2 (spontaneous deliveries), who presented with genital bleeding. She had asthma but had not received any medication for it. She had no previous history of obstetrics and gynecological issues. She had obtained a diploma in cooking and was a chef. She was from a family of three, and her economic situation was like that of most Japanese people. She was a tobacco smoker. Her family medical history was unremarkable. She was diagnosed as having cervical cancer, International Federation of Gynecology and Obstetrics (FIGO) clinical stage IB1. A histopathological examination was performed on a cervical biopsy sample, resulting in a diagnosis of squamous cell carcinoma. We explained that both surgery and radiation therapy are radical treatments; hence, she received radiation therapy with external irradiation (48 Gy × 24 times) and remote afterloading system (24 Gy × 4 times). Approximately 2 years after curative radiation therapy, she complained of itching, skin rash, and small papules and nodules on her left femoral and vulval skin (Fig. 1); these were edema and dermatitis flare-ups. A biopsy of an erythematous macule on her left femoral and vulval skin was performed. There were a few lymphocytes around the vessels, and a subsequent histopathological examination revealed cytokeratin-positive atypical cells invading the dermis; these were within the thin-walled vessels and expressed D2-40, a marker of lymphatic endothelium [13] (Fig. 2). The atypical cells were similar to the cells in squamous cell carcinoma. Positron emission tomography with computed tomography (PET-CT) revealed slight uptake of ^18^F-fluorodeoxyglucose (^18^F-FDG) in her left femoral skin with maximum standardized uptake value (SUV_max_) of 1.41 (Fig. 3). Apart from that, she had no other lesion with abnormal ^18^F-FDG uptake on PET-CT. She was subsequently diagnosed as having cutaneous lymphangitis carcinomatosa arising from cervical cancer. At the time of diagnosis, her muscle, circulatory, and respiratory functions were normal. She had normal blood pressure (129/69 mmHg), heart rate (71 beats per minute), and body temperature (36.9 °C) and did not have anemia (hemoglobin, 13.8 g/dL). Her liver and renal function were normal (total bilirubin, 0.5 mg/dL; aspartate aminotransferase, 19 U/L; alanine aminotransferase, 15 U/L; blood urea nitrogen, 13 mg/dL; and creatinine, 0.52 mg/dL), and her electrolyte levels were normal (sodium, 143 mEq/ml; potassium, 3.9 mEq/ml; and chloride, 109 mEq/ml). Moreover, her urine analysis results were normal (proteinuria, urinary glucose, and ketone were not detected). Paclitaxel (135 mg/m^2^, for 24 hours)-cisplatin (50 mg/m^2^, for 2 hours) and BV (15 mg/kg, for 1.5 hours) combination therapy was administered every 21 days. An improvement of both itching and rash was noted after three cycles. After a total of six cycles were administered, the femoral and vulval skin erythema completely disappeared (Fig. 4). Our patient experienced a symptom-free interval of 25 months after the last TP + BV infusion.

## Discussion

To the best of our knowledge, this is the first case report on TP + BV therapy for skin metastasis from cervical cancer. Skin metastasis occurs in 0.7–9% of all patients with cancer [14]. Skin metastases have the following distribution in women with primary malignancies: breast (69%), large intestine (9%), melanoma (5%), lung (4%), ovary (4%), sarcoma (2%), pancreas (2%), and uterine cervix (2%) [14]. The most common sites of skin metastases in patients with cervical cancer are the abdominal wall, vulva, anterior chest wall, and lower extremities [14]. Although skin metastasis mainly presents as nodules or masses, these lesions may sometimes appear as nodules or inflammatory disease [5, 14]. The morphologic patterns are nodules, plaques, and inflammatory telangiectatic lesions [6]. It is difficult to distinguish lymphangitis carcinomatosa from dermatitis. If the lesion is refractory dermatitis, a skin biopsy should be performed.

Currently, no effective treatment has been established for cutaneous metastasis due to lymphangitis carcinomatosa of cervical cancer [15, 16]. For recurrent cervical cancer, chemotherapy is one of the options available in Japan [17]. The results of the GOG 240 trial [9] suggested that TP + BV combination therapy can improve the prognosis of recurrent cervical cancer. Furthermore, in another study, the addition of BV to combination chemotherapy in patients with recurrent cervical cancer was associated with an improvement of 3.7 months in median overall survival [18]. There have been several reports describing the use of various regimens, including cisplatin-based chemotherapy and radiotherapy, in patients with recurrent skin metastasis from cervical cancer; however, most of these patients experienced progression of disease after chemotherapy (Table 1) [16, 19–25]. In this review, all skin metastases arose from squamous cell carcinomas. Only two cases advanced to stage III–IV, and eight cases were in stage I–II. Skin metastasis could arise from stage I–II cervical carcinoma. Skin metastasis from cervical cancer is possible even in the early stage. In addition to the present report, only one other case of complete response to chemotherapy has been reported, wherein the patient was treated with radiotherapy followed by chemotherapy [25]. Although palliative chemotherapy with paclitaxel resulted in complete clinical resolution in one previous case [23], to the best of our knowledge, this is the first report of complete response in a patient with skin metastasis of cervical cancer treated with combination chemotherapy plus BV.

**Table 1:** Literature for patients treated with chemotherapy against skin metastasis arising from squamous cervical carcinoma without other metastasis

Author/Year	Stage/Histology/Grade	Site of skin metastasis	Chemotherapy regime	Cycles	Best response
This report/2019	IB1/SCC/Differentiated	Thigh and vulva	PTX-CDDP-BV	6	CR
Özcan *et al*. [19]/2017	IB2/SCC/N/A	Vulva	1st line; PTX-CBDCA	2	PD
			2nd line; GEM-BV	N/A	PD
	IB1/SCC/N/A	Umbilicus (incisional scar), abdominal wall	1st line; PTX-CBDCA	1	N/A
			2nd line; GEM-BV + RT	1	N/A
Benoulaid *et al*. [20]/2016	IIIB/SCC/Moderately differentiated	Abdominal wall	CBDCA	4	PD
Basu and Mukherjee [21]/2013	IIA/SCC/Moderately differentiated	Thigh, inguinal region	CDDP-PTX + palliative RT	6	PR
Behtash *et al*. [16]/2008	IIB/SCC/N/A	Umbilicus	PTX-CBDCA	6	PR
Behtash *et al*. [22]/2002	IIA/SCC/N/A	Abdominal wall (drain site)	Cisplatin-5FU + palliative RT	6	PR
Palaia *et al*. [23]/2002	IIB/SCC/Poorly differentiated	Abdominal wall	Palliative chemotherapy (PTX)	10	CR
Kagen *et al*. [24]/2001	IB/SCC/Poorly differentiated	Thigh	Ifosfamide	N/A	PD
Freeman *et al*. [25]/1982	IVB/SCC/N/A	Abdominal wall	RT + Bleomycin-MTX -cyclophosphamide	N/A	CR

*5FU* 5 fluorouracil, *BV* bevacizumab, *CBDCA* carboplatin, *CDDP* cisplatin, *CR* complete response, *GEM* gemcitabine, *MTX* methotrexate, *N/A* not assessed, *PD* progressive disease, *PR* partial response, *PTX* paclitaxel, *RT* radiotherapy, *SCC* squamous cell carcinoma

In a mouse model of suture-induced corneal neovascularization, BV decreased cell proliferation of corneal lymphatic vessel cells through an anti-angiogenic effect [26]. Although the evidence supporting the anti-lymphangiogenic effects of BV in cancer is limited [27], BV has an antitumor effect in patients with breast cancer with lymph node metastasis [28]. Regarding lymphangitis carcinomatosa arising from other cancers, long survival has been reported in two cases treated with chemotherapy in combination with BV [29, 30]: paclitaxel and carboplatin (TC) in one patient with lung cancer and 5-fluorouracil, leucovorin, and oxaliplatin (mFOLFOX6) in a patient with colorectal cancer. Thus, BV may be more effective in metastases through lymph vessels, including lymphangitis carcinomatosa.

## Conclusion

In general, lymphangitis carcinomatosa is resistant to various therapies and has a poor prognosis. In the current case, TP + BV combination therapy was extremely effective against lymphangitis carcinomatosa. Our findings indicate that a chemotherapy regimen that includes bevacizumab should be considered an effective therapeutic option in patients with cutaneous lymphangitis carcinomatosa arising from cervical cancer.

## Data Availability

All data generated or analyzed during this study are included in this published article.
